# Rodent models of early adversity: Impacts on developing social behavior circuitry and clinical implications

**DOI:** 10.3389/fnbeh.2022.918862

**Published:** 2022-08-04

**Authors:** Katherine Packard, Maya Opendak

**Affiliations:** ^1^Department of Neuroscience, Kennedy Krieger Institute, Baltimore, MD, United States; ^2^Solomon H. Snyder Department of Neuroscience, The Johns Hopkins University School of Medicine, Baltimore, MD, United States

**Keywords:** early life stress, dopamine, social behavior, habenula, reward

## Abstract

Flexible and context-appropriate social functioning is key for survival across species. This flexibility also renders social behavior highly plastic, particularly during early development when attachment to caregiver can provide a template for future social processing. As a result, early caregiving adversity can have unique and lasting impacts on social behavior and even confer vulnerability to psychiatric disorders. However, the neural circuit mechanisms translating experience to outcome remain poorly understood. Here, we consider social behavior scaffolding through the lens of reward and threat processing. We begin by surveying several complementary rodent models of early adversity, which together have highlighted impacts on neural circuits processing social cues. We next explore these circuits underlying perturbed social functioning with focus on dopamine (DA) and its role in regions implicated in social and threat processing such as the prefrontal cortex (PFC), basolateral amygdala (BLA) and the lateral habenula (LHb). Finally, we turn to human populations once more to examine how altered DA signaling and LHb dysfunction may play a role in social anhedonia, a common feature in diagnoses such as schizophrenia and major depressive disorder (MDD). We argue that this translational focus is critical for identifying specific features of adversity that confer heightened vulnerability for clinical outcomes involving social cue processing.

## Introduction

For many species, appropriate social behavior is critical for access to key resources such as food, protection and receptive mates. The ability to accurately process and respond to social cues is particularly important during development, when environmental demands are in constant flux. For altricial mammals, social behavior in infancy is considered adaptive when it promotes approach toward the caregiver, the source of food, protection, and thermoregulation (Lorenz, 1966). This early social development also is occurring during a period of heightened plasticity in neural function, when environmental inputs can have lasting outcomes. As such, the early social environment is of key importance in programming lifelong social behavior patterns, particularly if this early environment is adverse and/or species-atypical (Tottenham, 2020). Recent approaches aimed at distinguishing dimensions of early adversity, in both humans and non-human animal models, provide a critically important tool for studying causation. Altogether, this body of work highlights the early social niche of the infant and perturbation of the caregiver-infant dyad in scaffolding neurobehavioral outcomes.

### Attachment as a framework for developing social behavior

The early social environment of the altricial infant is shaped by the caregiver and is perhaps best understood within the context of John Bowlby's Attachment Theory. Based on observations of imprinting in birds (Lorenz, 1966; Bowlby, 2007), this theory emphasizes how forming an emotional bond, or attachment, to a caregiver was an innate drive that promotes seeking proximity to a caregiver. This proximity-seeking ensures the altricial infant has access to key resources for survival (Bowlby, 1965, 1978). In addition, once the emotional attachment is formed, the caregiver gains unique access to regulation of the infant, including regulation of the infant's stress response (Hofer, 1973, 1994). This process, termed social buffering, has been observed across species, such as non-human primates, dogs and chicks (Hess, 1962; Stanley, 1962; Hinde and Spencer-Booth, 1970; Salzen, 1970; Rajecki et al., 1978; Lorenz, 1981), and is a feature of attachment relationships throughout the lifespan (Eisenberger et al., 2011; Hornstein and Eisenberger, 2017). In addition to regulating the infant's stress physiology, such as salivary cortisol (Hertsgaard et al., 1995; Hennessy et al., 2006; Taylor et al., 2008; Hostinar et al., 2015; Tottenham et al., 2019), recent studies in children have highlighted specific neural targets of regulation, such as infant cortical oscillations (Pratt et al., 2018), and functional connectivity between the medial prefrontal cortex and amygdala (Gee et al., 2014), regions critical for processing threat and fear. During a sensitive window in development, the presence of the caregiver can even impact whether children form an aversion or preference toward a previously neutral stimulus (Tottenham et al., 2019). Through this regulatory system, the altricial infant depends on their caregiver to know how to process cues in the environment. In this way, the infant's processing of the caregiver cue itself becomes of critical importance as both an early source of information and as a lasting template for social cue processing.

### Early care, attachment quality, and lifelong social behavior

Bowlby contended that the innate drive to seek proximity to a caregiver is strong enough to ensure an attachment is formed, regardless of the quality of care received. However, as emphasized in the work of the developmental psychologist Mary Ainsworth, adverse care conditions can impact the quality of attachments formed and the ability of the caregiver to regulate the infant (Ainsworth, 1969). This poor attachment quality is a robust predictor of lasting psychosocial function, though mechanisms remain unclear (Grady et al., 2016; Chambers, 2017; Granqvist et al., 2017; Keller, 2018; Vasileva and Petermann, 2018). However, it has been shown in children that poor quality attachments result in less effective social buffering of the infants' behavior and physiology (Nachmias et al., 1996), and decreased ability of the caregiver to impact the infant brain (Pratt et al., 2019). As will be discussed below, research manipulating early care environment in animals also results in poor caregiver regulation of the infant's neurophysiology, suggesting this is a promising translational channel for understanding the causal relationship between early caregiving environment, attachment quality, and psychosocial outcomes.

Decades of research on early care environment, such as work on children adopted from under-resourced orphanages, have highlighted impacts on nearly every level of neurobehavioral function, from epigenetics (Kumsta et al., 2016), to brain-wide neural network function (Malter Cohen et al., 2013; Callaghan et al., 2019), to social behavior (De Bellis and Thomas, 2003; Teicher et al., 2003; Nemeroff, 2004; Breslau, 2009; Tottenham et al., 2011; Carr et al., 2013; Stamoulis et al., 2015; Bryant, 2016; Zeanah and Sonuga-Barke, 2016; Heany et al., 2018; Vasileva and Petermann, 2018; Van Assche et al., 2019; Levis et al., 2021). Although these studies are highly informative about the outcomes of deprived early care, animal models permitting invasive approaches have provided a crucial tool for dissecting circuit mechanisms of early adversity impacts. Broad overviews of early life stress paradigms have been very effectively provided elsewhere and we direct readers to excellent examples of this work (Molet et al., 2014; Chen and Baram, 2015; Walker et al., 2017; Birnie et al., 2020; Wang et al., 2020; Levis et al., 2021). In the current mini-review, we limit our focus to three rodent models of early adversity that have been used to identify very specific impacts on infant processing of the caregiver *via* disruption of the midbrain dopamine system. These findings are then discussed within the context of clinical research on psychiatric disorders presenting with social behavior impairments and altered DA signaling, such as MDD and schizophrenia. We focus on the lateral habenula (LHb), as this is region is a negative regulator of the DA system and has been consistently implicated in both preclinical and clinical models of psychopathology, and argue that the animal models of early adversity may be useful in testing hypotheses generated by this clinical work.

## Animal models of early caregiving adversity: Approaches and findings

### Deprivation models

A powerful naturalistic adversity model is the paradigm of resource deprivation for mother rats and their newborn pups. Although there are many variations on this approach, including modeling neglect *via* repeatedly separating mother and pups (Hofer, 1973; Callaghan and Richardson, 2012; Tractenberg et al., 2016; Wang et al., 2020), our focus is on a common manipulation involving decreasing the amount of bedding in the cage during a sensitive window in the postpartum period (Pattwell and Bath, 2017; Walker et al., 2017; Goodwill et al., 2018). An exhaustive description of the results of these studies is beyond the scope of this article, although we direct the reader to excellent reviews on this topic, e.g., (Molet et al., 2014; Chen and Baram, 2015; Walker et al., 2017; Birnie et al., 2020; Wang et al., 2020; Levis et al., 2021). Here, we focus on a few key findings using the Scarcity-Adversity Model of Low Bedding (SAM-LB) (Roth and Sullivan, 2005; Rincón-Cortés and Sullivan, 2016). In this paradigm, mother rats are given decreased bedding materials with which to build a nest across several days, e.g., postnatal (PN) days 8–12. This results in increased rough handling, such as stepping on pups and dragging pups by the limbs. In this specific procedure, the cage has a solid floor and is not cleaned during the manipulation; this results in a milder approach than other low bedding paradigms, which can include a wire mesh floor. As a result, pups in the SAM-LB condition gain weight normally and reach typical developmental milestones (Opendak and Sullivan, 2019). However, this procedure results in increased plasma stress hormone levels in the pup and a robust and lasting effect on structure and function of key brain areas, circuits and molecules important for processing threat and social information (Raineki et al., 2019). Furthermore, this manipulation produces lasting deficits in socioaffective measures, such as social behavior, sleep, fear learning, and anxiety-like behavior (Raineki et al., 2012; Roth et al., 2014; Doherty et al., 2017; Santiago et al., 2018; Lewin et al., 2019). Importantly, findings using this procedure have many points of convergence with other early caregiving adversity models in rodents (Walker et al., 2017), non-human primates (Drury et al., 2015) and even children (Gunnar et al., 2015; VanTieghem and Tottenham, 2018; Callaghan et al., 2019; Tottenham, 2020). In particular, these cross-species studies highlight unique impacts of stress in a social context *via* neural circuits processing threat, safety, and caregiver cues (Callaghan et al., 2014, 2019; Opendak et al., 2020; Meyer et al., 2022).

Among the earliest emerging impacts of the SAM-LB manipulation is degraded ability of the caregiver to regulate the infant's neurobehavioral stress response (Shionoya et al., 2007; Moriceau et al., 2009). This can be uncovered by exposing infants to a mild aversive stimulus, such as a shock to the tail, either alone or with the mother (“Maternal Regulation Test”) (Opendak et al., 2021). Our group recently assessed this in pups that had undergone SAM-LB or control rearing from PN8–12 and were then given mild tail-shocks at PN13 (Opendak et al., 2021). In controls, the presence of the mother blocked shock-induced increases in pup plasma corticosterone, pup ultrasonic vocalizations, and pup motor activity. These effects were not observed in pups that had been shocked alone.

Additional studies have highlighted immediate changes to neural circuits and brain regions important for social buffering (Opendak et al., 2019; Robinson-Drummer et al., 2019). For example, in controls, maternal presence during classical Pavlovian odor-shock conditioning blocks associative learning and buffers plasticity in basolateral amygdala (BLA), a key region for processing threatening stimuli (Fanselow and LeDoux, 1999; Phelps and LeDoux, 2005; Johansen et al., 2011). On the circuit level, previous SAM-LB adversity prevented maternal buffering of the mesolimbic dopamine (DA) circuit, including the ventral tegmentum (VTA), nucleus accumbens, and BLA, as well as the interface between the medial prefrontal cortex and the BLA (Barr et al., 2009). Recently, research by our group has expanded this circuit to show that adversity also prevents the mother's ability to regulate the lateral habenula (LHb), an important negative regulator of the midbrain DA circuitry (Baker and Mizumori, 2017; Fakhoury, 2017; Hu et al., 2020, 2022; Germann et al., 2021; Nakamura et al., 2021; Packard et al., 2021).

As different brain regions mature at different rates, the timing of early adversity is a key consideration in designing experiments and interpreting neurobehavioral outcomes. Exposing pups to the SAM-LB manipulation from PN8–12 has been specifically shown to produce robust and lasting effects on numerous neurobehavioral outcomes, including fear learning, social behavior, and amygdala structure and function (Raineki et al., 2012, 2019; Walker et al., 2017). This time period is within the sensitive period for attachment and fear (e.g., before PN15), when circuitry supporting fear learning can be shut off by the presence of the caregiver (Moriceau and Sullivan, 2006). As such, heightened fear circuit plasticity, more dynamic stress hormone signaling, and reliance on the caregiver for buffering stress all render the infant rodent uniquely vulnerable to stress in a social context during this period (Sullivan and Holman, 2010).

Additional recent work using SAM-LB capitalizes on the ability of animal researchers to study what is occurring during the adversity itself, an impossible manipulation in humans. In this study, transmitters were implanted in the frontal cortex of pups for wireless telemetry recording of local field potentials (LFPs) (Opendak et al., 2020). Previous research had shown that in control rearing conditions, mere proximity to the mother produced a decrease in high frequency cortical oscillations, while nurturing inputs such as receiving milk and being groomed punctuated this general suppression with transient bursts in oscillatory power (Sarro et al., 2014). During SAM-LB, these same inputs failed to produce these expected changes, despite the fact that milk/grooming inputs occurred at the same frequency in both SAM-LB and control environments. This pattern of results paralleled findings observed using electroencephalography in children with impaired attachment to caregivers (Levy et al., 2017; Pratt et al., 2019). Finally, there was no difference in the cortical response to rough handling between conditions, the only maternal behavior to occur at a higher frequency in the SAM-LB condition. These surprising results suggest that the causal impact of SAM-LB may be through degraded cortical processing of *nurturing* inputs in a stressful environment, rather than adversity *per se*.

**Figure 1:**
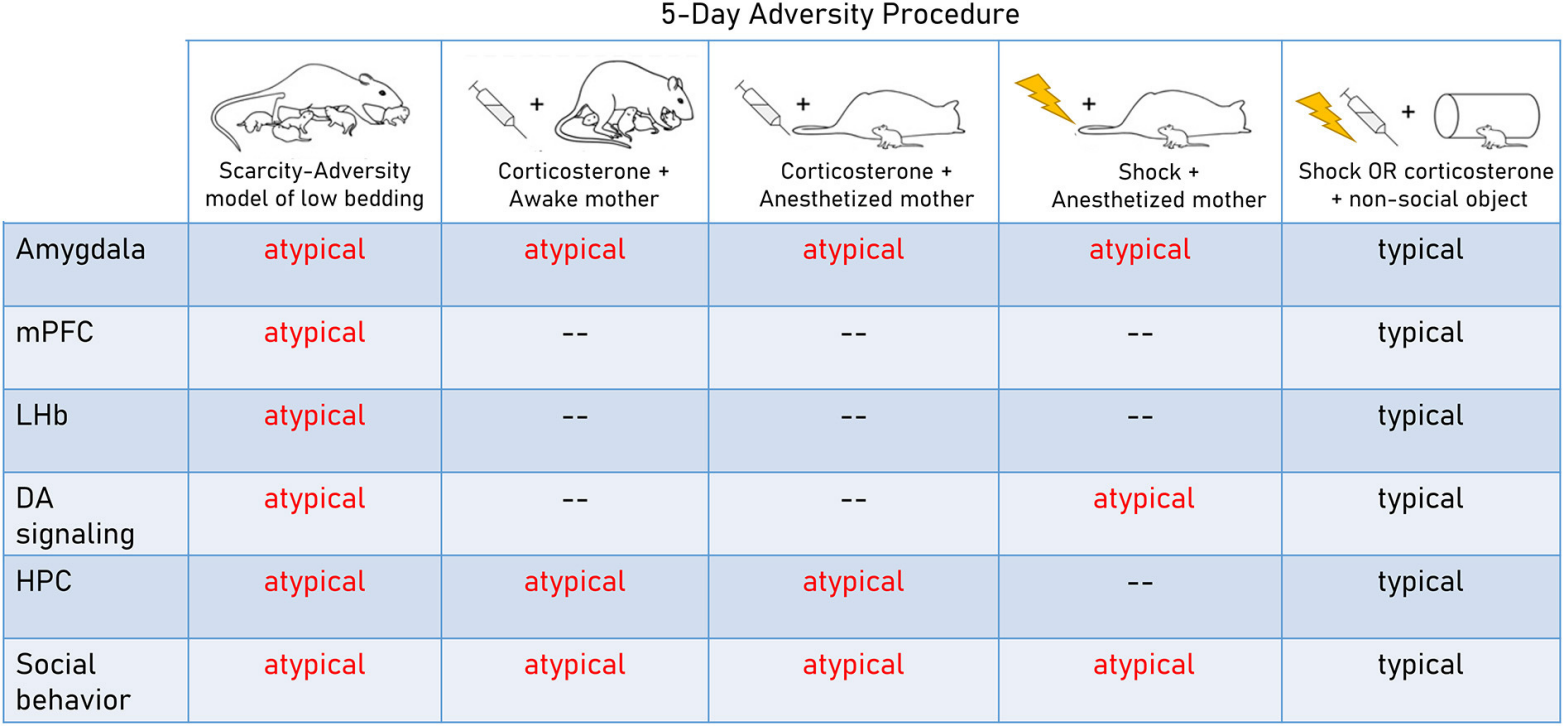
Neurobehavioral impacts of rodent adversity models in early life. Deconstructed and naturalistic adversity models highlight diffuse neural targets, including the amygdala interface with the dopamine system and the hippocampus. These studies show that repeated adversity across various social contexts and across various levels of naturalism/complexity is sufficient to produce deficits in social behavior and dysfunction in the amygdala. Importantly, atypical maternal behavior *per se* is not causal in producing these deficits. In contrast, adversity in a non-social context (repeated shock or corticosterone administration when pups are alone) is not sufficient to produce these outcomes (Bath et al., 2016; Rincón-Cortés and Sullivan, 2016; Walker et al., 2017; Goodwill et al., 2018; Perry et al., 2019; Raineki et al., 2019; Opendak et al., 2020, 2021).

A clear advantage of naturalistic approaches such as SAM-LB and other low bedding paradigms is their ethological relevance as well as translational relevance for work studying resource scarcity in humans. However, the implementation of these procedures varies widely across labs and naturally occurring variability in the treatment of individual pups within each litter is difficult to control (Walker et al., 2017). Therefore, deconstructed laboratory paradigms that permit researchers to isolate specific features of adversity are a useful complement to this approach and will be discussed below (summarized in Figure 1). These types of analyses have been increasingly applied in the developmental psychopathology field to determine which dimensions of early adversity drive maladaptive outcomes (e.g., Berman et al., 2022; Ellis et al., 2022; Nikolaidis et al., 2022).

### Deconstructed adversity procedure 1: Shock with mom vs. alone

Although the SAM-LB and other low bedding paradigms are informative, they leave several questions unanswered: Does the mother need to be the source of the rough treatment? Does the altered environment itself, e.g., altered bedding, drive impacts observed in pups? Would these same outcomes occur if painful rough handling was mimicked with a more controlled aversive stimulus, such as mild shocks? (Berman et al., 2022; Ellis et al., 2022; Nikolaidis et al., 2022). To begin addressing some of these questions, we recently presented a Deconstructed Adversity Procedure which permits researchers to isolate specific components of adversity to test their role in producing neurobehavioral outcomes. In this paradigm, pups are removed from the nest and receive repeated shocks to the tail each day during a sensitive period (e.g., PN8–12), either alone or in the presence of the dam (Opendak et al., 2021). The tail shocks are designed to mimic rough handling experienced during SAM-LB and are presented every 5 mins over a 45–90 min period each day. Unlike the Maternal Regulation Test described above, in which pup responses to acute shocks at e.g., PN13 are measured alone or with the dam, this Deconstructed procedure uses repeated shock alone or with the mother from PN8–12 as a tightly controlled form of adversity treatment. This procedure is a useful complement to the naturalistic procedure because it permits experimenters to ensure consistent treatment across subjects and perform invasive procedures to measure and manipulate brain function across the procedure that would be challenging in the naturalistic nest setting. Importantly, this approach can be used to dissociate the impacts of stress occurring in a social vs. non-social context.

Recent validation of this approach first showed that it was able to phenocopy the neurobehavioral impacts of SAM-LB on infant social buffering, fear network reactivity (e.g., BLA-VTA-mPFC), and social behavior (Opendak et al., 2021). Second, this approach permitted the experimenters to track individual pups over time to observe a gradual decrease in attachment behaviors toward the dam and gradual failure of the dam to buffer the neurobehavioral effects of the shock treatment. Furthermore, this approach permitted microdialysis and daily microinfusions during the adversity itself, which highlighted BLA DA release as a causal mechanism in degraded attachment behavior. Specifically, pups shocked with the mom showed an increase in BLA DA release, a reversal of the decrease observed in pups shocked alone. Furthermore, pups shocked repeatedly with the mother showed increased expression of DA receptor type 1 and tyrosine hydroxylase, the precursor to DA, in the BLA. These results set the stage for circuit manipulations using optogenetics that showed that persistent hyperactivity in the VTA-BLA circuit was causal in producing inhibited social approach behavior toward the mother and peers through PN23. Overall, these results highlighted that repeated adversity occurring in a social context produced distinct effects from adversity experienced alone, a critically important distinction for developing optimal therapeutics for individuals who have experienced trauma.

### Deconstructed adversity procedure 2: Corticosterone with mom vs. alone

Whereas the Shock-with-Mom procedure mimics the rough handling that pups experience in the Scarcity environment, another deconstructed approach involves simply injecting pups with the stress hormone corticosterone (CORT), either alone or with the mother (Raineki et al., 2019). This approach is a useful complement to the repeated shock procedure because it provides specific information about the effects of repeated stress on the neurohormonal level. In this paradigm, researchers repeatedly injected pups with either CORT or saline and then placed them with an awake, nurturing (control) mother, an anesthetized mother, or were placed alone in a chamber for 90 min daily from PN8–12. They found that all procedures involving repeated CORT injection, regardless of social context, resulted in structural impacts on the hippocampus (decreased neurogenesis, decreased volume). However, similar to results using a mild shock stressor, CORT injection repeatedly paired with a social context (awake, nurturing mother or anesthetized mother) resulted in decreased social approach toward the mother at PN13 as well as changes in amygdala structure and amygdala hyperactivity during social behavior testing. A key takeaway of these studies is that the effects of atypical or adverse maternal behavior, such as rough handling, can be mimicked by simply pairing repeated stress with maternal presence. These studies also showed that the amygdala is not typically involved in infant social behavior, and that early social adversity impairs social behavior through premature amygdala engagement. These latter findings are consistent with research in non-human primates (Amaral, 2003; Bachevalier and Loveland, 2006; Bliss-Moreau et al., 2011) and children who show heightened amygdala activity alongside inhibited social behavior (Monk et al., 2008; Tottenham et al., 2011).

Overall, these deconstructed approaches highlight the importance of the social context of early stress in producing social deficits and the role of early social buffering in scaffolding lasting pup social behavior. As such, these procedures provide an important complement to naturalistic manipulations in identifying the neurodevelopmental roots of social deficits.

## Early adversity, social behavior and psychopathology

A clear outcome that results from early adversity manipulations described in the previous section is that repeated social adversity degrades the salience of the caregiver cue. That is, the stimulus has a blunted impact on infant brain function and is less likely to elicit motivated approach behavior. Although the effects of early life stress are brain-wide (Saleh et al., 2017; Ohta et al., 2020), specific impacts on social information processing appear to occur *via* plasticity within the midbrain dopamine circuitry. This altered processing of social information may provide clues about the origins of social deficits observed in some psychiatric disorders. As will be discussed below, these neural circuits have been shown to be impacted in individuals with clinical disorders, and early adversity confers an increased risk for developing psychopathology *via* impacts on these circuits (Philip et al., 2013; VanTieghem and Tottenham, 2017).

Although the brain's social behavior circuitry is complex and numerous brain areas are impacted in disordered social processing, here, we turn our focus to studies of upstream control of DA signaling by the LHb. LHb dysfunction is one of the most robust correlates of adult psychopathology across preclinical and clinical models (Fakhoury, 2017; Yang et al., 2018; Barreiros et al., 2022; Mondoloni et al., 2022), yet causal experiments that leverage animal models of early adversity to study developmental impacts on this system are limited (Nakamura et al., 2021; Langlois et al., 2022). Below, we summarize some of the key clinical evidence supporting a focus on this area in future work on the pathogenesis of disordered social processing.

### Social anhedonia in clinical populations: Involvement of dopamine

Convergent findings in both human and animal models suggests that early social adversity weakens the impact of social inputs on the developing brain (Gunnar et al., 2015; Callaghan et al., 2019; Pratt et al., 2019; Opendak et al., 2020). This effect may provide an early risk factor for the clinical phenomenon of **social anhedonia**, a disinterest and inability to find pleasure in social interactions (Ritsner et al., 2018). In humans, social anhedonia is found in many diagnoses including schizophrenia and MDD and can result in lack of social support (Weeks et al., 1980; Llerena et al., 2012; Eglit et al., 2018). With social support being identified as a predictor of good prognosis and quality of life, blunted social functioning proves to be a major obstacle for those with mental health diagnoses (Chevallier et al., 2012; Eglit et al., 2018; Sagud et al., 2021).

### Social anhedonia, reward processing and the habenula

**Figure 2:**
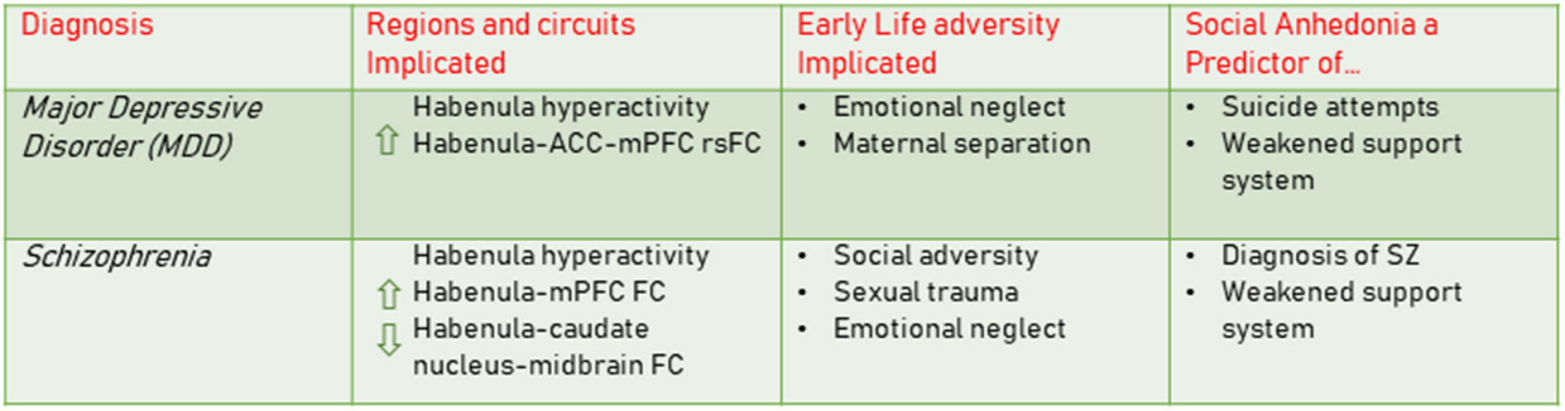
MDD and Schizophrenia: Links to habenula dysfunction, early life adversity, and social anhedonia. The clinical literature has demonstrated links between MDD and schizophrenia and habenula dysfunction, including hyperactivity and its connectivity with other brain regions (Shepard et al., 2006; Proulx et al., 2014; Fakhoury, 2017; Zhang et al., 2017; Barreiros et al., 2022). Early adversities such as emotional neglect, maternal separation and sexual trauma are contributing factors conferring increased risk for both of these disorders (Kessler et al., 2010; Bentall et al., 2012; Heim and Binder, 2012; Akdeniz et al., 2014; Wickham and Bentall, 2016). Social anhedonia is a key feature of these disorders and impacts the individual's ability to form a strong social support system. This symptom can be predictive of suicide attempts in MDD and is an early emerging predictor of a schizophrenia diagnosis (Cohen et al., 2020). MDD, major depressive disorder; SZ, schizophrenia; FC, functional connectivity; rsFC, resting state functional connectivity; mPFC, medial prefrontal cortex; ACC, anterior cingulate cortex.

Many diagnoses involving anhedonia can be examined through the lens of motivation and reward and the role of dopamine signaling. However, less is known linking upstream habenula function, early adversity, and social dysfunction in clinical outcomes. We therefore focus our current discussion on the habenula and the robust evidence for its role in the development and/or exacerbation of social processing deficits in MDD and schizophrenia (summarized in Figure 2). We argue that this framework provides a strong rationale for further research on mechanisms of habenula dysfunction using the deconstructed early adversity models described above. As it is often difficult to differentiate between subregions of the habenula in fMRI work, we use the term habenula while exploring the human literature.

As described above, the habenula is a negative circuit regulator of midbrain DA release *via* the VTA (Proulx et al., 2014) and plays a key role in processing negative valence information, the absence of expected rewards, and supporting learned aversions (Yang et al., 2018). The habenula has also been shown to respond to punishment and negative feedback (Shepard et al., 2006), which are important cues for adaptive behavior modification. However, habenula activity beyond the normative range may lead to motivation deficits and hypervigilance in avoiding aversive situations beyond what is necessary for survival – a potential factor conferring vulnerability in psychiatric disorders. Indeed, resting-state fMRI data showed structural differences of the habenula in those with schizophrenia and MDD relative to healthy controls (Proulx et al., 2014; Liu et al., 2017; Browne et al., 2018) as well as differences in functional connectivity between habenula and regions such as the mPFC (Zhang et al., 2017) (Figure 2).

### Habenular dopamine suppression in major depressive disorder and schizophrenia

Beyond these basic differences, habenular hyperactivity has impacts for reward processing and social functioning outcomes for those with MDD and schizophrenia. Chronic DA suppression from an overactive habenula may contribute to anhedonia and persistent feelings of an aversive state, a potential contributor to MDD (Dichter, 2010; American Psychiatric Association, 2013; Proulx et al., 2014). Recent work links general anhedonia with increased suicidal ideation, and social anhedonia with increased suicide attempts (Sagud et al., 2021). Furthermore, recent work has shown increased habenular activity in response to feedback in those with MDD in addition to blunted reward prediction error and increased punishment prediction error correlating with depressive episodes, implicating perturbed reward learning in MDD (Liu et al., 2017; Kumar et al., 2018). Finally, deep brain stimulation suppressing the habenula (thus reversing chronic inhibition of DA neurons) has shown promising results in reversal of depressive symptoms in treatment-resistant patients (Proulx et al., 2014; Browne et al., 2018; Yang et al., 2018). These results suggest that one mechanistic aspect of MDD may be failed reward learning, wherein habenular hyperactivity causes failure to learn rewards and biases toward anticipating punishment over reward.

In schizophrenia, habenular processing of negative stimuli to promote behavior adjustments and aversion learning can also go awry. fMRI data has shown lessened ability to adjust behavior in response to task feedback in those with schizophrenia (Shepard et al., 2006). Additionally, this study showed diminished concurrent activity between the habenula and midbrain in response to negative feedback and between the habenula and caudate nucleus in response to positive feedback (Shepard et al., 2006). Among many functions, these regions are known to contribute to motivation and learning, respectively. Furthermore, habenular dysfunction may contribute to difficulties in assigning reward values to stimuli (Fakhoury, 2017), which may play a role in social anhedonia which is common in schizophrenia, and even predictive of the diagnosis (Eglit et al., 2018; Cohen et al., 2020). Together, these findings highlight possible mechanistic links between habenular dysfunction, social reward processing, and clinical outcomes.

Importantly, the risks for developing disorders such as MDD and schizophrenia are increased following early life adversity (ELA) (Kessler et al., 2010; Akdeniz et al., 2014; Hoffmann et al., 2017). Although ELA impacts are brain-wide, the clinical literature has shown convergence with animal research demonstrating effects on regions processing threat and reward described above (Meyer et al., 2022). Examples of specific connections between adult MDD and ELA have been shown with respect to reward circuit function (Hanson et al., 2021) as well as amygdala and medial prefrontal cortical volume abnormalities (Heim and Binder, 2012; Shepard and Nugent, 2021). Studies have also highlighted the importance of the type and severity of adversity in producing these outcomes. For example, chronic HPA activation in children increases the risk for developing both MDD and schizophrenia (Smart et al., 2015; Popovic et al., 2019; Silva et al., 2021) and dose-response effects have been shown between childhood adversity and later life depressive disorders (Chapman et al., 2004). Furthermore, ELA occurring in a social context has been shown to increase the risk for later-life psychosis (Bentall et al., 2012), highlighting the salience of adversity experienced in the context of a social influences such as the caregiver. Recent work further supporting adversity's role in the development of psychosis links early life sexual abuse to hallucinations, and early life emotional neglect with paranoia (Wickham and Bentall, 2016). Finally, dose-response effects of early trauma on later life psychosis have been identified, suggesting ELA plays a causal role in psychosis (Varese et al., 2012). Taken together, these data suggest mechanisms by which certain types of ELA may interact with developing monoaminergic circuitry, and the lasting clinical implications of this perturbation.

## Concluding thoughts

Maladaptive and inflexible social behavior is an early-emerging symptom of numerous psychiatric disorders, such as MDD and schizophrenia. Although the pathogenesis of these disorders is unclear, converging evidence indicates that individual vulnerability to developing these pathologies is heightened by early adversity, particularly in the context of atypical caregiving quality. However, it has been challenging to identify the mechanisms translating this early experience to later social behavior outcomes.

Here, we have summarized key evidence from human and non-human animal research suggesting that this perturbed early social environment impacts social behavior through alterations in processing social cues. Together, these studies highlight impacts on the midbrain dopamine system and its projections to/from brain areas important for processing threat and reward, including the amygdala, prefrontal cortex, and lateral habenula. We have focused on very specific early life adversity manipulations which provide key information dissociating the impacts on these circuits of stress alone from those of stress occurring in a social context. These paradigms comprise only a small sample of the numerous informative early adversity procedures that are available to researchers, and are themselves limited in their translational applications. However, we argue that these tightly-controlled experimental approaches provide a unique opportunity for testing circuit hypotheses generated by the clinical literature. Future cross-species research leveraging deconstructed early adversity paradigms will be critical for identifying causal links between early risk factors and specific loci of dysfunction in psychiatric disorders.

## Author contributions

KP and MO wrote the review. Both authors contributed to the article and approved the submitted version.

## Funding

The authors would like to thank NIH for funding of this research (BRAIN R00MH124434) and the Brain and Behavior Research Foundation (Young Investigator Grant to MO).

## Conflict of interest

The authors declare that the research was conducted in the absence of any commercial or financial relationships that could be construed as a potential conflict of interest.

## Publisher's note

All claims expressed in this article are solely those of the authors and do not necessarily represent those of their affiliated organizations, or those of the publisher, the editors and the reviewers. Any product that may be evaluated in this article, or claim that may be made by its manufacturer, is not guaranteed or endorsed by the publisher.
